# Machine-Learning Ice Spectra: From 1 to 256 Features

**DOI:** 10.1021/acs.jctc.5c01413

**Published:** 2026-02-04

**Authors:** Shokirbek Shermukhamedov, Jolla Kullgren, Daniel Sethio, Kersti Hermansson

**Affiliations:** Department of Chemistry-Ångström, 8097 Uppsala University, Box 538, Uppsala S-75231, Sweden

## Abstract

The study explores how well machine learning and structural fingerprints can predict spectroscopic properties of ice (OH vibrational frequencies and ^1^H chemical shifts). A large theoretical data set (55 ice polymorphs, 1010 DFT data points both for the vibrations and for the NMR shifts) and a smaller cross-validation set are employed. The Message Passing Atomic Cluster Expansion (MACE) model performs the best, with high accuracy (root-mean-square deviation, RMSD, of 0.06 ppm for chemical shifts and ∼10 cm^–1^ for vibrational frequencies). Simpler descriptors like ACSF and SOAP, when paired with suitable regressors, nearly match MACE’s performance. At the other end of the complexity scale, it is found that using the simplest possible physics-based descriptor of the environment (a single H-bond distance) yields RMSD values three times as large for the vibrations and four times as large for the proton chemical shift compared to the MACE model. Depending on the context, those RMSD values may still be considered modest and useful, considering the gain in simplicity and transparency.

## Graphical abstract

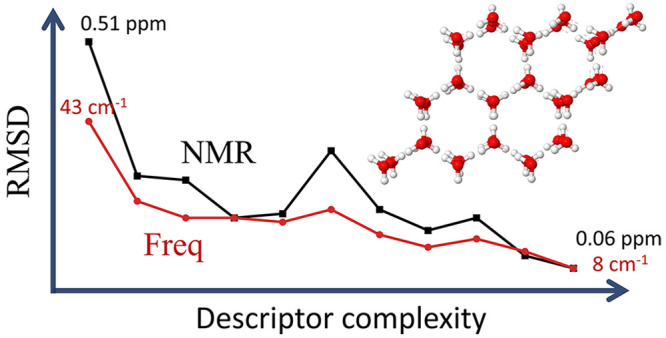

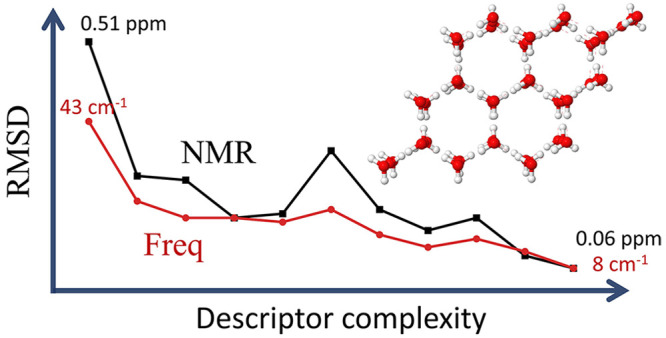

## 1 Introduction

This study deals with machine-learning (ML) prediction of spectroscopic properties, namely nuclear magnetic resonance (NMR) chemical shifts and vibrational frequencies. These represent some of the most powerful techniques for determining the local atomic-level structures in molecular condensed matter. Here, with access to two theoretical ice data sets, one of them very large, we use ML techniques to compare how well it is possible to predict the OH vibrational frequencies and the proton chemical shifts, using a range of structural descriptors (and some regressors). Or differently put: we examine how sensitive these spectroscopic properties are to different structural features (descriptors) of the surroundings.

Ice polymorphs constitute an interesting testbed for such a comparison as, on the one hand, their compositions are outstandingly simple, and the structures are seemingly similar and uncomplicated, except for the large variations in density among the polymorphs. On the other hand, ices are challenging systems to model and master as long-range intermolecular interactions are appreciable, the water molecule being both polar and polarizable.

For molecular condensed matter in general, there have been some considerable efforts in the literature to design workflows that achieve stand-alone prediction of NMR spectra with the ultimate goal of replacing experiments, or at least providing reasonable proxies for experimental spectra; see e.g., the review article by Jonas et al.^1^ One example of such methodologies is ShiftML,^2,3^ which uses machine-learning models trained on large data sets of chemical shielding values from density functional theory (DFT) calculations of organic crystals and with local structural descriptors based on smooth overlap of atomic positions (SOAP^4^). In a ShiftML study by Cordova et al.,^2^ chemical shielding values for ∼14,000 molecular crystal structures were used to train spectroscopic models for crystals containing several different elements. The resulting root-mean-square deviation (RMSD) between the predicted ^1^H chemical shifts and the DFT reference values came out to be about 0.50 ppm. Using Δ-machine learning and atom-centered descriptors, Unzueta et al. reached an RMSD value of 0.11 ppm for a test set of diverse medium-sized organic molecules containing H, C, N and O.^5^

For ML-aided prediction of vibrational frequencies, one useful approach is to construct machine-learning potentials, which are then used in molecular dynamics simulations to yield power spectra, or alternatively, Raman/IR/SFG intensity-weighted spectra; see, e.g., refs 6 and 7. The approach taken in our paper is different, as we make ML predictions directly on the vibrational frequencies. Such approaches are less common in the literature although several examples already exist. One example is the work of Raimbault et al.,^42^ who trained Raman frequencies and polarizabilities on DFTMD data for one crystalline form of paracetamol and predicted the Raman spectra of another crystalline form (polymorph) with good accuracy. Another example is the work of Kananenka et al.^8^ who trained ML models for frequencies and dipole moment derivatives to predict the infrared OH stretching band in liquid water. Also for liquid water, Kwac et al.^9^ generated models for three target properties: OH frequencies, IR intensities and Raman intensities. For the frequencies, the lowest RMSD value (33 cm^–1^) was reached with a descriptor based on the atom-centered symmetry functions (ACSF).^10^

Also in the current study we generate predictive models for spectral signatures from quantum-mechanical DFT data, more precisely based on data sets of ice structures. The largest data set represents 1100 unique ^1^H isotropic chemical shifts and 1100 unique OH vibrational frequencies from many ice polymorphs. We obtain an RMSD value of 0.06 ppm for the δ^1^H chemical shift and about 10 cm^–1^ for the ν­(OH) vibrational frequency for our best “ML descriptor”, namely MACE.^11^ The goal of the present work is, however, not only to produce target models with the best possible predictive power, but we will also discuss the value of models which sacrifice some predictive power for either model evaluation speed or model interpretability.

Important methodological aspects of our study are the following.

(i) We compare vibrational and NMR spectroscopies with respect to how OH frequencies and ^1^H chemical shifts respond to, or capture, the same set of increasingly elaborate structural features (expressed by our chosen descriptors).
(ii) Our descriptors only consider the intermolecular surroundings of the OH oscillator or the NMR-shielded proton on each targeted water molecule, i.e., we omit the intramolecular structural parameters although including such information is likely to improve the fitting quality.
(iii) To achieve as consistent comparisons between descriptors as possible, we use the same structures, the same DFT method, consistent hyperparameters, and the same measures of quality in all comparisons, both for the vibrational and the NMR models.
(iv) We also use a second training philosophy for the comparison, namely one where we fine-tune the hyperparameters to achieve the “best possible” prediction with each descriptor.
(v) We make use of two—seemingly rather homogeneous—data sets of ice structures, where the starting geometries were taken from the literature. The structural data sets were curated by us to ensure the uniqueness of each entry, and the structures were subsequently reoptimized with one selected vdW-inclusive DFT functional, which was also used for the spectral calculations. Both standard fitting and further cross-validation analyses were undertaken. The latter adds useful information as an essential characteristic of any predictive model is its ability to generalize to unseen data, often referred to as extrapolation capability.
(vi) OH vibrations are strongly anharmonic (important for OH groups), and we include nuclear quantum effects by solving a quantum vibrational Hamiltonian. Furthermore, we do not use any scaling or shifting of any of the calculated quantities presented in this report.

It is useful to discuss the RMSDs resulting from our computational predictions in the light of the attainable experimental precisions. The most accurate experimental water OH vibrational spectra in the literature for crystalline hydrates originate from low-temperature, isotope-isolated experiments and yield well resolved spectra with OH frequencies determined to a precision of 5–10 cm^–1^ or better.^12^ For the ices, we have not found isotope-isolated vibrational experiments in the literature, but one example of a quite well resolved 100% H_2_O spectrum is the Raman study of Ice Ih in ref 13. Turning to NMR chemical shifts, ^1^H chemical shifts from solid-state Magic Angle Spinning NMR measurements can generally be measured to a high precision. The publication guidelines for the ACS journals recommend reporting ^1^H NMR chemical shifts in ppm to two digits after the decimal point. However, again, for ice and supercooled water, it is not easy to find benchmark values. A study of supercooled nanoconfined water^14^ presented static NMR experiments at ambient pressure in the temperature interval 195 K < *T* < 293 K and reported an uncertainty of ±0.05 ppm for the chemical shift values. We hypothesize that high-quality experimental OH vibrational frequencies and ^1^H chemical shift measurements for well-behaved ices under favorable conditions may be as good as 5–10 cm^–1^ and 0.02–0.05 ppm, respectively.

In this paper we will find out if our computational-based predictions can match these uncertainties.

The organization of the paper is as follows. Systems, data sets and methods are reported in Section 2, concluding remarks are given in Section 5, and in between lies Section 3 (Results and Discussion), which is arranged as follows: Section 3.1 Predictability of Different Descriptors, Section 3.2 Descriptor Fine-Tuning (using the GPR regressor), Section 3.3 Extrapolation Capabilities of the Models, Section 3.4 Data Set Size, and Section 4 which summarizes our findings from Sections 3.1–3.4.

## 2 Data Sets and Methods

The initial structures were taken from two structural databases, namely that of Nanayakkara et al. (ref 15, referred to as the Kraka structures in the following) and the Materials Project.^16^ They were further curated by us as described in Section 2.1, and were subsequently reoptimized by us using DFT at the optPBE-vdW level.^17^ Anharmonic O–H vibrational frequencies and NMR chemical shieldings of the protons were calculated with the same DFT method for the two structural data sets, and constitute our spectroscopic data sets used for training, validation and testing. Section 2.2 gives an overview of the characteristics of the two structural data sets. All DFT calculations are described in Section 2.3. Technical details concerning the model training and validation are given in Section 2.4.

### 2.1 The Ice Structures

Table 1 lists some main features of our DFT-generated ice data sets. The data sets are labeled “Kraka-UU” and “MP-UU” to highlight both the origin of the starting structures and the fact that they have undergone data set curation and subsequent structural reoptimizations at the optPBE-vdW level by us. We will often drop the “–UU” suffix in the text, tables and figures for simplicity; thus wherever we write “Kraka” or “Kraka-UU” in our Results tables and figures they both refer to the curated structures at their optPBE-vdW-optimized values, and similarly for the MP data.

**Table 1:** Characteristics of Our Curated and Reoptimized Ice Data Sets^a^

Structural data set	No. of structures	No. of H atoms	No of unique H atoms	R(O···O) range (Å)	R(H···O) range (Å)	ν(OH) range (cm^–1^)	δ^1^H range (ppm)
Kraka-UU	55	1788	1010	2.72–2.97	1.72–1.98	2865–3230	–22.8 to −26.1
MP-UU	10	244	43	2.67–3.59	1.73–2.02	2840–3180	–22.6 to –25.8

a The ^1^H chemical shift is denoted δ^1^H and the anharmonic OH vibrational frequency is denoted ν­(OH).

Strictly speaking we are dealing with six data sets: the Kraka-UU structures, NMR data for the Kraka-UU structures, ν­(OH) data for the Kraka-UU structures, the MP-UU structures, NMR data for the MP-UU structures, and ν­(OH) data for the MP-UU structures. It will be clear from the context which data we refer to. Section S1 of the Supporting Information provides more details regarding the starting structures and the curation processes for both the Kraka-UU and MP-UU data sets.

#### 2.1.1 The Kraka-UU Ice Structures

The published ice data set of Nanayakkara et al.^15^ consists of structures for 55 ice polymorphs. For those structures that have been deemed as being disordered in experimental studies, Nanayakkara et al. created ordered variants using the GenIce algorithm,^18^ which generates random hydrogen-bond networks, while maintaining four-coordination around each water molecule with two donated and two accepted H-bonds per molecule as well as a zero dipole moment of the crystallographic unit cell. In this way, they generated a large number of ordered alternatives for each disordered polymorph. All were geometry-optimized by them using the vdW-DF2 density functional method, and a handful of the most stable structures for each disordered polymorph were kept for further analysis and also saved in a repository which we made use of here. Regarding experimentally ordered ice polymorphs, only one such structure is present in the repository of Nanayakkara et al.

Altogether their data set contains 1788 hydrogen atoms, but many of them are symmetry-equivalents in agreement with the space group symmetries. The redundant (symmetry-equivalent) copies were removed by us with the help of the Pymatgen program,^19,20^ leaving 1010 structurally unique H atoms, i.e., 1010 OH groups and 1010 ^1^H nuclei with structurally unique environments. The structures were reoptimized by us at the optPBE-vdW level, as mentioned, and the Kraka-UU structural information in Table 1 refers to those reoptimized structures.

#### 2.1.2 The MP-UU Ice Structures

The Materials Project (MP) database^16^ was searched by us for structures with the sum formula H_2_O (with no other elements present). Sixteen such structures were found. Most of these structures represent different ice polymorphs. The ice structures in the MP are all optimized at the PBE level, with starting structures taken from various sources, some of them experimental structures, others corresponding to artificial structures. We found that a few of the MP structures with sum formula “H_2_O” contain hydrogen atoms located midway between two oxygen atoms while in some other cases, H is located unphysically close to an oxygen atom. Structures displaying such motifs were omitted from our dataset. Furthermore, one of the structures was found to contain dissociated water molecules, also this structure was omitted. After this curation of the MP data set, we were left with a data set of 10 MP structures. All of these are structurally different from the Kraka data set structures which we verified using Pymatgen.^20^

Altogether these 10 ice structures contain 244 hydrogen atoms, but many of them are symmetry-equivalents in agreement with the space group symmetries. The redundant (symmetry-equivalent) copies were removed by us with the help of the Pymatgen program.^20^ Our final MP-UU data set contains 43 structurally unique H atoms. The structures were reoptimized by us at the optPBE-vdW level, as mentioned, and the MP-UU structural information in Table 1 refers to those repotimized structures.

### 2.2 Characterization of Our Structures

The spectroscopic properties were calculated as will be described in Section 2.3. Among structural features, hydrogen-bond distances are of course of particular interest and two of the most commonly quoted H-bond distances in aqueous systems in the literature are R­(O···O) and R­(H···O), defined in Figure 1d. The ranges of R­(O···O), R­(H···O), δ^1^H, and ν­(OH) for the Kraka-UU and MP-UUdata are given in Table 1 and the latter three are also displayed in Figure 1a,c,e as probability density functions (pdfs). Figure 1b,d highlights that the data points from our MP-UU data set are located “within” that of the Kraka-UU dataset. This is corroborated by the PCA analysis of the MACE feature space presented in Figure S1.

**Figure 1:**
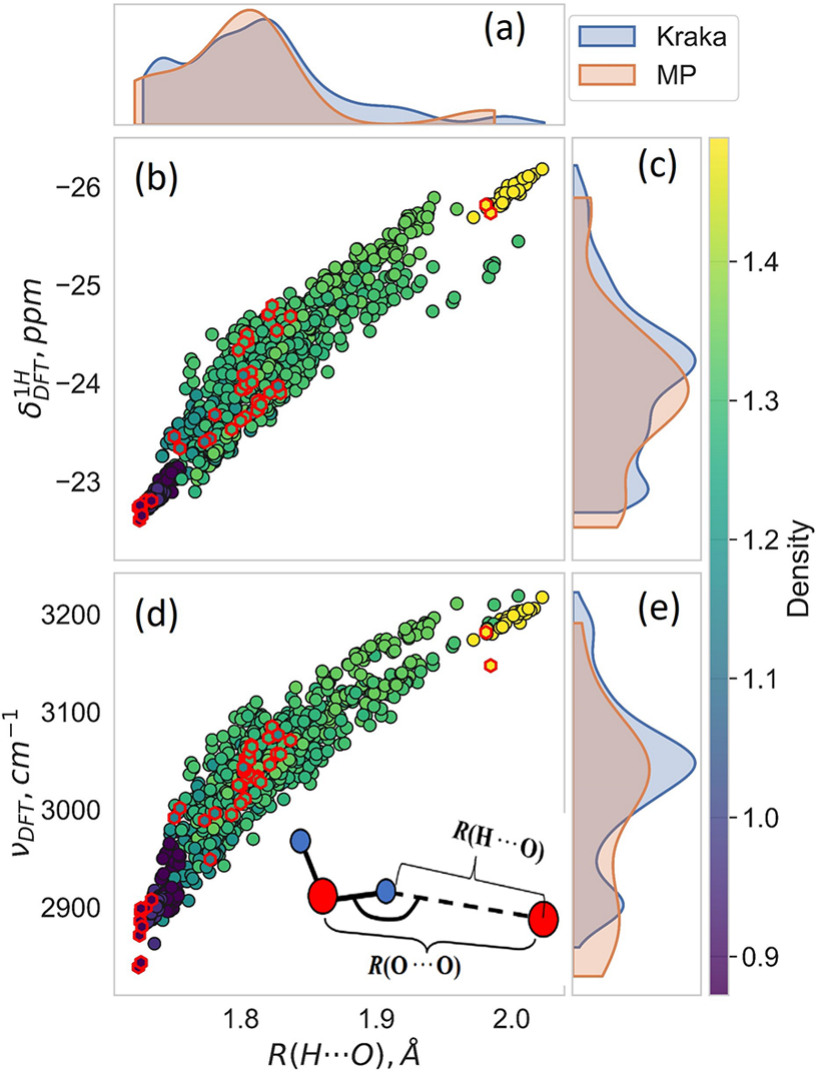
Characterization of the optPBE-vdW DFT data sets used in this study (referred to as Kraka or Kraka-UU data interchangeably, and similarly for MP and MP-UU). The figure visualizes the data presented in Table 1. Panels (a), (c) and (e) at the top and right-hand edges of the figure display the distributions and boundaries of our DFT-calculated R­(H···O) distances, ν­(OH) vibrational frequencies, and δ^1^H NMR chemical shifts, respectively, for the two ice data sets. Panels (b) and (d) give the ‘ν­(OH) vs R­(H···O)’ and ‘δ^1^H vs R­(H···O)’ correlation curves for all H atoms in the data sets. The Kraka-UU data are represented by black-rimmed filled rings, with color indicating the calculated crystal density (g/cm^3^) (cf. the color bar along the rightmost edge of the figure). Similarly, red-rimmed hexagons represent the MP-UU data, also color-coded by density. In both panels, the rings/hexagons in the lower left-hand corner correspond to those H atoms that are involved in the strongest H-bonds, while the H atoms in the upper right-hand corner are weakly H-bonded. The schematic inset in panel (d) illustrates the definitions of the two types of H-bond distances most commonly used in the literature to characterise OH···O hydrogen bonding.

To eliminate remaining reduncancies in our data sets we only keep one replica of a frequency or NMR value originating from the same crystal structure if they are equal after rounding off to one or two decimal places, respectively. The calculated densities of the various energy-optimized polymorphs range from 0.83 to 1.44 g/cm^3^ in the Kraka-UU data set and from 0.96 to 1.48 g/cm^3^ in the MP-UU data set.

#### 2.2.1 Definition of a Hydrogen Bond

In this paper we define a hydrogen bond as existing when R­(H···O) < 2.5 Å and the angle­(O–H···O) > 120°.

### 2.3 DFT Calculations of Structures, Vibrational Frequencies, and NMR Chemical Shifts

#### 2.3.1 Electronic Structure Calculations and Optimized Structures

The optPBE-vdW functional was used in this study, as we have earlier^21,22^ performed some extensive tests with this functional for crystalline hydrates, with respect to both structure and vibrational frequency. Calculations were performed with the VASP 6.3.3 software,^23^ employing the PAW method with “hard” pseudopotentials.^24,25^ Key settings included a 1000 eV plane-wave cutoff, a self-consistent field convergence threshold of 10^–7^ eV, a k-spacing of 0.33 Å^–1^, and a Gaussian smearing of 0.1 eV. Spin polarization was omitted. Structure optimization was carried out until the forces on each atom and the stress were less than 10^–7^ eV/Å and 10^–7^ kbar, respectively.

#### 2.3.2 OH Frequencies, ν­(OH)

In vibrational spectroscopy, the frequency and intensities of an oscillating atom, or group of atoms, serve as probes of their surroundings. Here we will only be concerned with the frequencies, as this already is a sensitive and much used probe of the surroundings of a molecular oscillator. Similarly to the optimized structures, the OH stretching vibrational frequencies were obtained from periodic calculations. For each unique O–H group in each ice crystal, a one-dimensional uncoupled vibrational model was used to map the anharmonic potential energy curve *in situ* in the crystal. The vibrational problem was solved using 1-dimensional quantum dynamics. Thus, our vibrational model uses high precision in one dimension and neglects multidimensionality effects. This is intentional because the aim is to mimic isotope-isolated experiments, which experimentally are superior to all-H (or all-D) experiments both regarding peak resolution, and regarding the possibility to interpret the spectral signals in terms of features of the surrounding structure. In an experimental isotope-isolated experiment, the OH stretching mode essentially becomes decoupled from its environment.

In the literature one finds that a fair number of experimentalists go through this extra work of isotope-isolation in order to get rid of the vibrational couplings (and similarly regarding spin–spin couplings in NMR). In an earlier computational study,^26^ we assessed to what extent our 1-D vibrational approach yields results in line with isotope-isolated experiments (as simulated by us). Those simulated experiments were performed by means of a set of full lattice dynamics calculations, where in turn only one of the H atoms was assigned the mass of ^1^H and all others were ^2^H, and then this was repeated consecutively for all hydrogens in the cell. The test confirmed the almost perfect OH vibrational decoupling and the adequacy of the 1-D approach (Figure 5 in ref 26).

To solve the anharmonic oscillator problem, the O–H bond was stretched and compressed at 71 points around its equilibrium position, while the rest of the system was held fixed. The vibrational Schrödinger equation was solved using the discrete variable representation (DVR^27^), providing vibrational eigenvalues for analysis. The fundamental vibrational wavenumber was calculated from the energy difference between the ground state and the first excited state (a more elaborate description of the current methodology is given by Mitev et al.^26^).

#### 2.3.3 The Proton Chemical Shift, δ^1^H

The NMR chemical shift calculations were performed at the same level of theory as the energy minimizations and the frequency calculations. The LCHIMAG routine in VASP was used to generate the isotropic chemical shielding coefficient σ^iso^, which is obtained from the trace of the chemical shielding tensor. Finally, the isotropic chemical shift, δ^1^H, is obtained from the δ^iso^ = σ^iso^_ref_ – σ^iso^_sample_ expression, where σ^iso^_ref_ refers to a selected reference system. The δ^1^H values in the figures and tables are those originating directly from the VASP output, which by default assumes a σ^iso^_ref_ value of zero, i.e., **δ** ^**1**^H is the negative of the σ^iso^_sample_ value. The VASP output values for δ^1^H are thus negative. When a water molecule becomes involved in H-bonding to other water molecules the chemical shieldings of its protons diminish, i.e., become less positive, and the chemical shift values in the VASP output become less negative. This is consistent with the ‘**δ** ^**1**^H vs H-bond’ distance plot in Figure 1b.

Incidentally, our calculated shielding value for H in the gas-phase water molecule is 30.58 ppm, corresponding to a δ^1^H value of −30.58 ppm.

We do not consider magnetic couplings between nearby proton spins. Thus, similarly to our approach for the vibrational frequencies, also here our approach mimics isotope-diluted experiments, which makes it easier—both experimentally and computationally—to decipher the influences from the local environment on each probed ^1^H nucleus without interference from couplings with other intra- and intermolecular spins.

### 2.4 Model Building—Descriptors and Training

With the four spectral data sets—ν­(OH) from Kraka-UU, δ^1^H from Kraka-UU, ν­(OH) from MP-UU, δ^1^H from MP-UU—we performed model training and explored the impacts of different descriptors and regressors on the predictability of our ν­(OH) and δ^1^H models. Some technical details are given in this section.

#### 2.4.1 Descriptors

All descriptors used in this paper are structural (“geometrical”) in nature. We have loosely categorized our descriptors as distance-based descriptors, atom-centered descriptors (with and without angular terms) and a graph neural network. In detail we use the following descriptors: single distances R­(O···O) or R­(H···O), multidistance features, atom-centered descriptors [namely: Smooth Overlap of Atomic Positions (SOAP),^31^ Pair Distribution Functions (PDF),^32^ Local Many-body Tensor Representation (LMBTR),^33^ Atom-centered Symmetry Functions,^10^ a weighted version of ACSF (wACSF),^34^] and the machine learning-based MACE (Message Passing Atomic Cluster Expansion) descriptor. The descriptors, except MACE, were calculated using the ASE,^35^ Scikit-learn,^36^ DScribe^37^ and n2p2 ^38^ packages. All descriptors used in the fitting of model functions are defined at their appropriate places in the Results and Discussion section.

For MACE, the model features were taken from the MACE-MP(0) model^11,39^ and used *as is*. It consists of 10 radial basis functions to encode radial information.^39^ The angular component of the MACE model is represented by spherical harmonics with *l*_max_ = 3. To ensure a fair comparison across different descriptors, we generated features aligned with the hyperparameters-settings of the MACE model for all models. For this purpose, a cutoff radius of 6 Å was always used. Thus, the atom-centered descriptors used radial grids, ranging from 0 to 6 Å with 10 bins, and including angular information. Such a “MACE-flavored” set of descriptors will be referred to as a “*MACE-consistent descriptors*” in Chapter 3 and the results will be described in Section 3.1.

We have also explored the limits of the various descriptor’s prediction capabilities when the (hyper)­parameters are set to yield the smallest possible loss. We label the set of descriptors with such settings “*fine-tuned descriptors*”. The results are given in Section 3.2. Note that, also in these explorations, we have kept the cutoff radius fixed at 6 Å for all descriptors, where applicable.

#### 2.4.2 Only External Structure Included in the Descriptor

When constructing the descriptors, then for each targeted H atom we discarded the O and the other H atom in the targeted water molecule. Thus, we eliminated information about the intramolecular geometry from the description of the targeted H atom’s environment as we want to focus solely on prediction of the effect of “the water molecule’s *inter*molecular surroundings” on ν­(OH) and δ^1^H. Or differently put, this way the *“structural fingerprint → ν­(OH) and δ* ^1^ *H*” correlations can be used in the opposite direction to yield information about the external surroundings of a bound molecule, which is in fact often the reason for performing spectroscopic measurements in the first place.

#### 2.4.3 Training

For each descriptor, we fitted model functions (f: $R^{N}\to R$ , where N is the number of features in the descriptor) using two groups of regression methods: linear models and probabilistic ones. The linear models included Linear Regression, Ridge Regression and Bayesian-Ridge (BR) regression, while the probabilistic methods comprised Gaussian Process Regression (GPR) using a dot-product + white noise kernel, as well as a dot-product kernel with an exponentiation of 4, as we earlier did in ref. 22. The fitting was made using the Scikit-learn^36^ package. Additionally, we explored neural network (NN)-based architectures, including multilayer perceptrons (MLP), convolutional neural networks (CNN), and recurrent neural networks, particularly Long Short-Term Memory (LSTM) networks, and those were fitted using the Keras package.^40^

#### 2.4.4 Metrics Used for Model Assessment

The prediction capability will be assessed both by validation within the same data set (“*Kraka* → *Kraka*” and “*MP* → *MP”*) and by cross-validation between the Kraka and MP data sets (“*Kraka* → *MP*” and “*MP* → *Kraka*”); see definition of notation in Table 2. The quality of the ν­(OH) and δ^1^H values from our trained models compared to the DFT-calculated reference values will be assessed using the root-mean-square-deviation (RMSD) score, the goodness-of-fit (R^2^) values, and absolute maximum deviation (AMD) values. All were calculated from 5-fold splits of the training data which corresponds to an 80:20 splitting.

**Table 2:** Definition of Labels That We Use in the Tables and Text to Identify the Data Sets Involved in This Study as Well as Their Roles in the ML Model Creation and Its Assessment, i e., as Training, Validation, Testing

**Label** ^a^	Means...
*Kraka* → *Kraka*	The ν(OH) or δ^1^H model is trained on Kraka-UU data. The listed RMSDs etc are calculated on the “validation data sets” obtained from 5-fold splits of the Kraka-UU data.
*Kraka* → *MP*	Cross-validation: The ν(OH) or δ^1^H model is trained on Kraka-UU data but the listed RMSDs etc are those resulting when the model is applied to the MP-UU data (the “test set”).
*MP* → *MP*	The ν(OH) or δ^1^H model is trained on MP-UU data. The listed RMSDs etc are calculated on the “validation data sets” obtained from 5-fold splits of the MP-UU data.
*MP* → *Kraka*	Cross-validation: The ν(OH) or δ^1^H model is trained on MP-UU data but the listed RMSDs etc are those resulting when the model is applied to the Kraka-UU data set (the “test set”).

a “Kraka” data always means “Kraka-UU” data. “MP” data always means “MP-UU” data.

## 3 Results and Discussion

In this chapter we explore the performance of a range of descriptor/regressor combinations for the vibrational and NMR data and compare the results. Performance here primarily refers to the prediction ability of the models. But not only. Other assets that can make a model competitive will also be discussed such as extrapolation ability, efficiency (use cost), and a model’s ability to provide physical insight. As mentioned, in Section 3.1 we work with a set of “MACE-consistent” descriptors where, for example, all use a cutoff of 6 Å (except the smallest distance-based descriptors which by virtue of their nature have much shorter cutoffs; see further the Method section). Such an approach, in addition to the fact that we use the same data points in all trainings, the same regressor (GPR), the same measures of quality (RMSD etc.) allows for a consistent comparison between descriptors and between the NMR and vibrational data. In Section 3.2, on the other hand, we fine-tune the descriptors and address how accurately we can fit to the DFT data if we aim for the best possible hyperparameters for each of the descriptor types and regression models. In Section 3.3 we discuss the extrapolation capabilities of the models and in Section 3.4 the predictive capabilities under different data set sizes. Section 4 summarizes our findings from Chapter 5.

### 3.1 Predictability of Different Descriptors

We start by choosing one particular descriptor as an illustration of how we will examine and assess our spectral models. Our sample descriptor here is the simplest, and perhaps the most intuitive, structural descriptor for a hydrogen-bonded system, namely R­(H···O).

Figure 2 shows *correlation* and *scatter plots,* respectively, involving R­(H···O) and the two spectroscopic signals over the Kraka-UU data set (often called “Kraka” dataset in the following, as pointed out above). The data points in Figure 2a,d constitute *correlation plots* for “ν_DFT_ or δ^1^H_DFT_ versus R_DFT‑opt_(H···O)”, i.e., all data come directly from our optPBE-vdW calculations. GPR fittings were subsequently performed on the spectroscopic data with a dot-product kernel with exponent 4 and using R­(H···O) as the structural descriptor. Figure 2b shows a scatter plot for ‘predicted vs DFT’ frequencies when R­(H···O) is used as a descriptor, and similarly in Figure 2e for the NMR chemical shifts. The orange curves drawn in Figures 2a,d are representative model functions generated by the same models whose scatter plots are shown in Figures 2b, e. Those model curves are superposed on the ‘ν_DFT_ vs R­(H···O)_DFT_’ and ‘δ­(^1^H)_DFT_ vs R­(H···O)_DFT_’ correlation distributions in Figures 2a and d, respectively. The reference DFT vibrational frequencies and NMR chemical shifts in Figure 2a,d are seen to essentially form continuous distributions and the representative model curve drawn in each of the two panels manages quite well to capture the DFT-based data; they are seen to form a one-to-one function.

**Figure 2:**
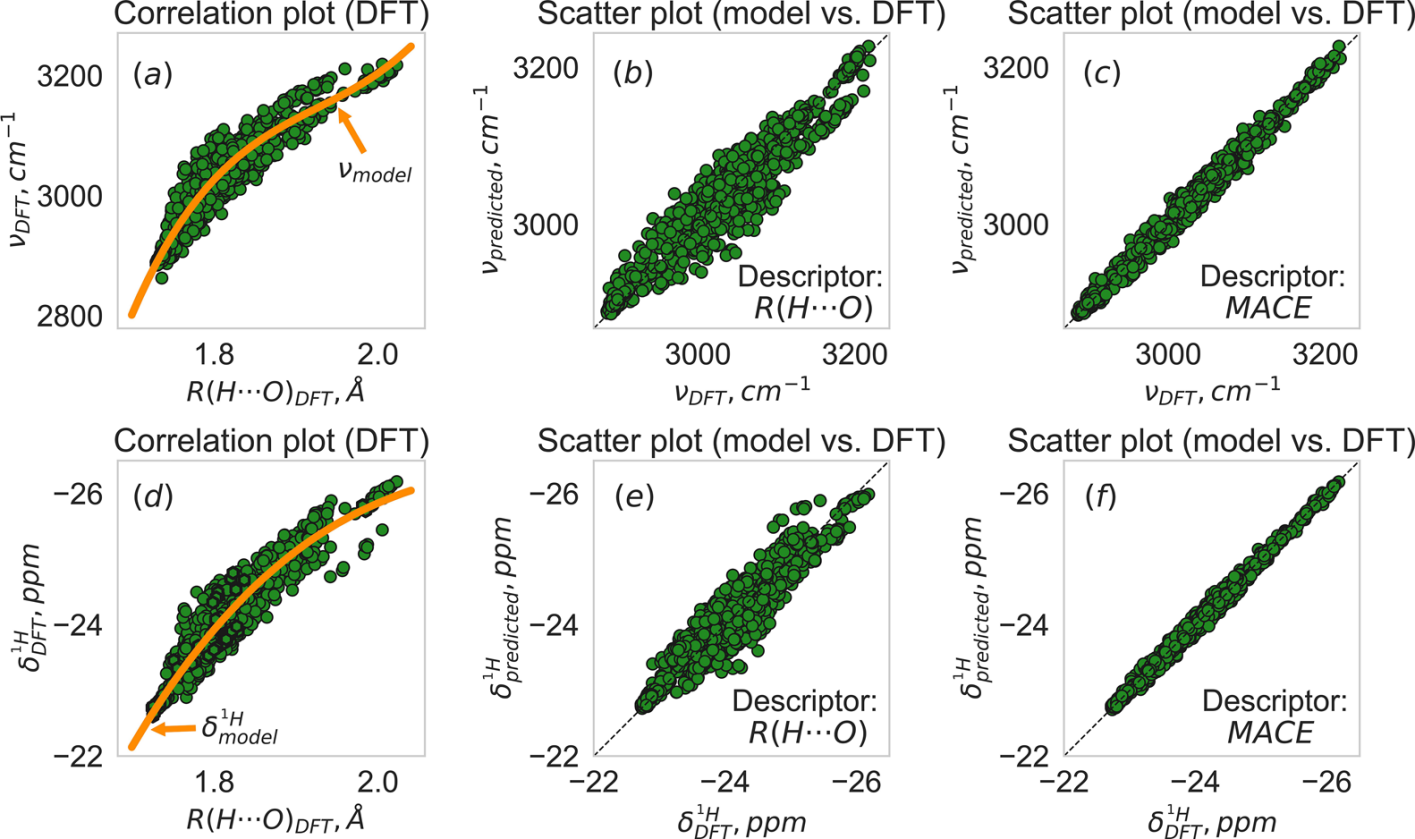
Examples of the graphs that we use in relation to data analysis and model generation in this study; all are based on the Kraka-UU data in this figure. The upper row refers to ν­(OH), the bottom row to δ^1^H. The left-hand column shows correlation plots between DFT-generated properties. Scatter plots of predicted model values vs DFT reference values are shown in the middle and right-hand columns, using two different descriptors: (b), (e) use R­(H···O); (c), (f) use MACE. The orange curves, marked ν_model_ in (a) and (d), are representative model curves from the GPR regressions in (b) and (e), respectively, i.e., using the R­(H···O) descriptor.

The average RMSD values calculated over the Kraka data set are 23 cm^–1^ and 0.24 ppm, respectively. Figure 2c,f highlight what the scatter plots look like for the most predictive models that we have achieved (namely using the MACE descriptor).

In the following, we will provide a detailed analysis of each descriptor’s prediction performance in order of increasing complexity of the descriptors. The complexity of descriptors ranges from simple pairwise distances like R­(H···O) and R­(O···O), through multidistance features (tetrahedron, R30­(H···X), R30­(H···O)), distribution-based (PDF, LMBTR) and symmetry-functions-based models (ACSF/wACSF), to the SOAP descriptor, and finally the MACE neural network-based descriptor, which offers the most expressive but computationally demanding and least interpretable representation. Our underlying question is: *How accurately can our model functions describe our complex spectroscopic data*?

Table 3 lists information about the type and feature size of each descriptor, along with the performance metrics. In this table we use the MACE-consistent descriptor approach. As seen in the table, the MACE descriptor, with a feature size of 256, gives the most accurate predictions, for both ν­(OH) and δ^1^H. The resulting scatter plots (predicted vs reference values) were already shown in Figure 2. The progressions of the RMSD values as a function of feature size are displayed in Table 3 and Figure 3. Not surprisingly perhaps, for descriptors with only a small number of features the nature of the features is crucial. Here much can be gained in terms of physical insight by choosing adequate descriptors. In the following we will discuss the descriptor performance from the top to the bottom in Table 3, which is roughly, but not exactly, the same as from left to right in Figure 3, since as just said, the nature of the descriptor features matters, not just the number of them. In all cases in Figure 3 and Table 3 we used the GPR regressor with dot product and white kernels.

**Table 3:** Fittings Using “MACE-Consistent Descriptors”^a^

			OH frequency with GPR Kraka → Kraka^b^			^1^H chemical shift with GPR Kraka → Kraka^b^		
Type of structural descriptor	Descriptor	Feature length	RMSD^c^ (cm^–1^)	*R* ^2^	AMD (cm^–1^)	RMSD^c^(ppm)	*R* ^2^	AMD (ppm)
Distance-based	R(O···O)	1	62	0.30	104	0.54	0.08	0.91
	R(H···O)	1	30	0.84	91	0.28	0.87	0.82
	Tetrahedron	4	29	0.86	79	0.25	0.90	0.70
	R30(H···X)	30	21	0.92	58	0.21	0.86	0.72
	R30(H···O)	30	20	0.93	50	0.20	0.88	0.44
Radial-based + angular	PDF	10	36	0.77	105	0.41	0.46	1.15
	LMBTR	58	22	0.91	66	0.27	0.77	0.86
	ACSF	38	17	0.95	55	0.16	0.92	0.48
	wACSF	16	20	0.93	62	0.18	0.89	0.57
Radial + sph. harmonics	SOAP	840	11	0.98	42	0.07	0.98	0.29
Graph neural network	MACE	256	8	0.99	36	0.06	0.99	0.19

a Prediction capability of the various descriptors used in this work. In this table, the models were all trained on the Kraka data set with the GPR regressor and also validated on the Kraka data set. MACE-consistent hyperparameters were used; see the text for further information Here and in the following, the performance metrics of the models are reported as averages over 5-fold splits. RMSD = root-mean-square deviation, *R* ^2^ = goodness-of-fit (a value between 0 and 1, where 1 is the best), AMD = absolute maximum deviation. See the text for more information about descriptors and feature lengths. In this table, the results were obtained with MACE-consistent hyperparameters; see the text for further information.

b The key for the A → B notation is given in Table 2.

c The RMSD values for the OH frequencies and ^1^H chemical shifts are given to precisions of 1 cm^–1^ and 0.01 ppm in the tables, guided by the fact that their ranges are large: about 390 cm^–1^ and 3.5 ppm, respectively, as seen in Table 1.

**Figure 3:**
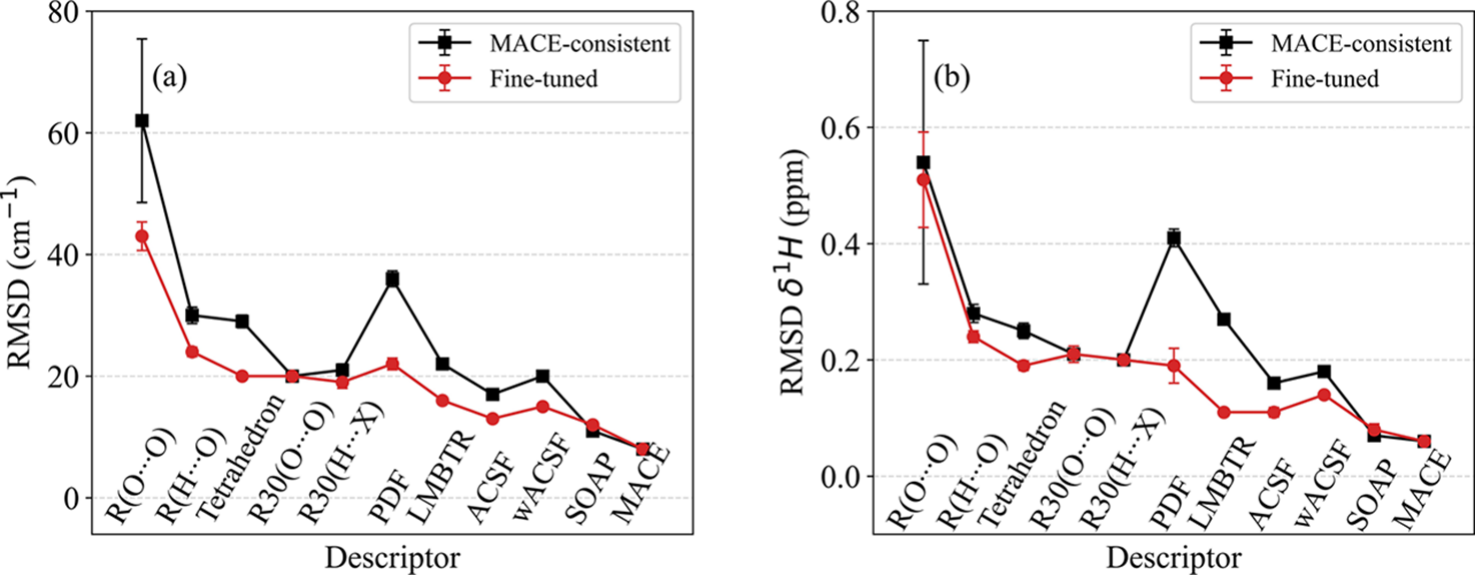
RMSD values of OH frequency (a) and NMR (b) values obtained from 5-fold validation within the Kraka data set (“Kraka → Kraka” values in Tables 3 and 4) plotted in order of increasing descriptor complexity. (Black line) “MACE-consistent” descriptor settings, (Red line) fine-tuned descriptors with optimal regressor. For each descriptor, the standard deviation of the RMSDs across the 5-fold splits is shown as error bars and is only appreciable for the R­(O···O) descriptor.

#### 3.1.1 Distance-Based Descriptors

Five types of distance-based descriptors were assessed with respect to their ability to capture the total influence of the crystalline surroundings on ν­(OH) and δ^1^H. (1) **R­(O···O)**: The distance between the H-bond donating O atom (bound to the targeted H atom) and the acceptor oxygen atoms in the surroundings (cf. Figure 1d). (2) **R­(H···O)**: The distance between the targeted H atom and its H-bond-accepting oxygen atom (cf. Figure 1d). (3) In the **tetrahedron** descriptor, inspired by Pfrommer et al.^41^ but without their internal geometry information, we use all hydrogen bonds that are donated to, or accepted by, the water molecule in question. The distances are sorted such that the first distance corresponds to R­(H···O), the second distance is the other hydrogen bond donated by the water in questions. The last two distances are the two hydrogen bonds accepted by the water molecule in question. (4) **R30­(H···X)**: Distances between the targeted hydrogen atom and its 30 nearest neighbor atoms, within the cutoff radius of *r*_cutoff_ = 6 Å. (5) **R30­(H···O)**: Distances between the targeted H atom and its 30 nearest oxygen atoms within the same *r*_cutoff_ = 6 Å. As shown in Table 3, R­(H···O) yields RMSD value of 30 cm^–1^ for vibrational frequencies and performs rather well also for NMR predictions (0.28 ppm). In contrast, R­(O···O) yields unsatisfactory results for both properties. The tetrahedron descriptor slightly improves frequency predictions compared to R­(H···O), while NMR prediction improves by about 10%. Including more neighbors in the fitting process improves prediction quality further, with R30­(H···O) performing slightly better than R30­(H···X).

#### 3.1.2 Atom-Centered Descriptors

This group of descriptors transforms the local atomic environment of each atom, using distances and angles to neighboring atoms to capture geometric and chemical information. In our study, this category includes two related subgroups of descriptors: PDF/LMBTR and ACSF/wACSF. The first group uses pairwise distance distribution as the radial part. PDF does not include any angular information, whereas LMBTR incorporates angular information between atoms into the feature vector. The second group uses a combination of radial and angular symmetry functions based on exponential functions that encode interatomic distances and angles to ensure invariance to translation, rotation, and permutation. Moreover, the LMBTR and ACSF descriptors are computed for each pair of elements, resulting in vector spaces that separately capture the H_target_–H and H_target_–O environments. In contrast, PDF and wACSF consider the entire environment: PDF counts the presence of atoms while ignoring their types, and wACSF counts atom types by assigning weights, thus, in principle, preserving more information from the local atomic environment.

The ACSF/wACSF group of descriptors provide a more detailed description of the surroundings but becomes computationally expensive as the number of elements in the system increases. This is also true for LMBTR. PDF, on the other hand, is more general and simpler but less accurate, making it suitable for more general prediction tasks. To match MACE hyperparameters we used 10 radial bins with cutoff 6 Å for the first group (and angular terms for LMBTR) and 10 symmetry functions and 3 pairs of angular symmetry functions for the second group. Among these descriptors, ACSF performs the best, with RMSD values of 17 cm^–1^ and 0.16 ppm. However, these results are significantly worse than those obtained with SOAP. The LMBTR and wACSF descriptors show an accuracy level comparable to the distance-based ones, while PDF performs the worst.

#### 3.1.3 SOAP Descriptors

The SOAP descriptor describes the local atomic environment around a given atom by expanding the neighbor density in terms of radial basis functions (RBFs) and spherical harmonics functions. This yields a fingerprint of the local atomic environment that is invariant under rotation and translation. The table shows that the SOAP descriptor can fit both vibrational frequencies and NMR values with high accuracy, much better than the distance-based descriptors presented above. However, with RBF = 10 and *l*_max_ = 3 the number of features reaches 840, i.e., resulting in a large feature vector.

#### 3.1.4 MACE

The MACE model used here is a graph neural network-based foundation model designed to serve as a universal force field for inorganic compounds.^39^ Here it is used as a descriptor and is seen to demonstrate superior performance for our two spectral properties, achieving RMSD values of 8 cm^–1^ for vibrational frequency predictions and 0.06 ppm for the NMR property. The R^2^ scores are nearly perfect at 0.99 for both ν­(OH) and δ^1^H, indicating a near-linear relationship between predictions and target values (cf. Figure 2c,f). However, the descriptor exhibits rather high AMD values, highlighting areas for potential refinement.

#### 3.1.5 Conclusions Concerning the MACE-Consistent-Descriptor Comparison

Overall, MACE emerges as the best-performing descriptor, with the lowest metric scores among all tested descriptors. Its ability to effectively capture the local atomic environment makes it a powerful tool for modeling complex systems. We also note that the simple R­(H···O) descriptor performs rather well.

### 3.2 Descriptor Fine-Tuning

Above we selected descriptor hyperparameters (i.e., those parameters that are not fitted in our regressions) to match the conditions used for the MACE descriptor used here. However, each descriptor in our list exhibits distinct behavior with respect to its hyperparameters. For example, in the case of the ACSF descriptor, we initially used 10 radial symmetry functions but increased the number to 32 as increasing this hyperparameter affects the accuracy but was found to not significantly affect the computational time required to compute the features. We also performed additional calculations to find optimal settings for each atom-centered descriptor; for details see Section S3. Table 4 and Figure 3 display the results, which can readily be compared with the “MACE-consistent” hyperparameters presented above. It should be noted that now the feature lengths of all descriptors apart from the distance-based descriptors and MACE are results from our optimization of the hyperparameters. The standard deviation of the RMSD across the 5-fold validation is indicated in Figure 3 and is only appreciable for the R­(O···O) descriptor.

**Table 4:** Fittings Using Fine-Tuned Descriptors^a^

				OH frequency with GPR Kraka → Kraka			^1^H chemical shift with GPR Kraka → Kraka		
Type of structural descriptor	Descriptor	Regressor	Feature length	RMSD (cm^–1^)	*R* ^2^	AMD (cm^–1^)	RMSD (ppm)	*R* ^2^	AMD (ppm)
Distance-based	R(O···O)	GPR(exp)	1	43	0.67	195	0.51	0.16	1.34
	R(H···O)	GPR(exp)	1	23	0.91	76	0.24	0.90	0.75
	Tetrahedron	GPR(exp)	4	20	0.93	67	0.19	0.93	0.69
	R30(H···X)	GPR(wd)/BR	30	19	0.92	58	0.21	0.86	0.72
	R30(H···O)	GPR(wd)/BR	30	20	0.93	50	0.20	0.88	0.44
Radial-based + angular	PDF	GPR(wd)/BR	200	22	0.91	77	0.19	0.88	0.59
	LMBTR	GPR(wd)/BR	148	16	0.96	56	0.11	0.96	0.36
	ACSF	GPR(wd)/BR	94	14	0.97	46	0.11	0.96	0.34
	wACSF	GPR(wd)/BR	42	16	0.96	56	0.14	0.94	0.45
Radial + sph. harmonics	SOAP	GPR(wd)	144	11	0.98	42	0.08	0.98	0.30
Graph neural network	MACE	GPR(wd)/BR	256	8	0.99	36	0.06	0.99	0.19

a Performance metrics for the same descriptors as in Table 3, but here the optimal combination of GPR kernels and fine tuned descriptors is used in each case. GPR stands for Gaussian process regressor with exponential (Exp) or “white kernel + dot product kernel” (wd). GPR­(wd)/BR means that GPR­(wd) and BR perform equally well. In the next two tables, the results were obtained with our best hyperparameters, i.e., the fine-tuned hyperparameters.

PDF and LMBTR descriptors have two main hyperparameters: the number of radial grid points (and also angular grid points for LMBTR) and the scaling factor. We find that increasing the number of radial grid points decreases the RMSD values; however, using 50 radial bins and a scaling factor of 0.1 is sufficient to achieve valuable results for the LMBTR. The PDF descriptor shows about a 30% improvement in performance by fine-tuning.

For ACSF and wACSF, the initially applied combination of 10 radial and 3 pairs of angular functions proved to be suboptimal. Lower RMSD values were achieved with 32 radial and 5 pairs of angular functions. Slight improvement is observed when the angular function pairs are increased to 9.

Additionally, the radial and angular components of the ACSF descriptor can be considered independently. In the corresponding tables in the SI, the first row (marked as 0) presents results using only the radial part of the descriptors. For frequency predictions, using only the radial components provides accuracy comparable to that of the distance-based descriptors R30­(H···X) and R30­(H···O), whereas including angular information leads to improved accuracy for the NMR predictions.

Since the radial part of the wACSF descriptor alone provides acceptable accuracy, the addition of angular functions leads to only a modest improvement in performance. This suggests that, for wACSF, most of the relevant structural information is already captured through radial distances and the weighting by atom type.

The MACE-consistent SOAP hyperparameters already deliver near-perfect results (Table 3). However, the resulting number of features is extremely large. A practical strategy to address this is reducing the feature vector length while maintaining prediction accuracy. Optimization of the SOAP hyperparameters shows that lowering the number of radial basis functions to 4 reduces the number of features by a factor of 4, with only a minimal impact on performance. It should be noted that descriptor fine-tuning was performed without a separate test set and followed the same approach and conditions as in the MACE-consistent calculations.

### 3.3 Extrapolation Capabilities of the Models

In this section we assess extrapolation by performing cross-validation using the Kraka and MP data sets. Specifically, we present two complementary schemes: in one case, we used the Kraka data set for training and the MP data set for testing (Kraka → MP), while in the other, the roles of the data sets were reversed, i.e., training on the MP data set and testing on the Kraka data set (MP → Kraka). These computer experiments allow us to rigorously assess the performance of our model functions and their ability to predict properties outside the scope of the training data.

Table 5 presents the RMSD values for frequency predictions using the GPR model across two data sets, highlighting the impact of data set size on model performance. The results demonstrate that training on a more extensive data set (Kraka) effectively captures information relevant to the smaller data set (MP), as presented in the scatter plots for the MACE descriptor in Figure 2c,f.

**Table 5:** RMSD Values for Cross-Validation Calculations, Using Optimal Combinations of GPR Kernels and Fine-Tuned Descriptors

	OH frequency RMSD (cm^–1^)				^1^H chemical shift RMSD (ppm)			
	Data sets				Data sets			
Descriptor ↓	Kraka → Kraka	Kraka → MP	MP → MP	MP → Kraka	Kraka → Kraka	Kraka → MP	MP → MP	MP → Kraka
R(O···O)	43	59	56	67	0.51	0.45	0.40	0.62
R(H···O)	23	37	46	33	0.24	0.27	0.40	0.60
Tetrahedron	20	21	38	37	0.19	0.31	0.32	0.28
R30(H···X)	19	23	62	80	0.21	0.21	0.36	0.72
R30(H···O)	20	22	92	102	0.20	0.18	0.24	0.57
PDF	22	22	72	146	0.19	0.24	0.29	0.89
LMBTR	16	20	39	73	0.11	0.11	0.28	0.81
ACSF	14	16	51	94	0.11	0.12	0.21	0.57
wACSF	16	17	124	171	0.14	0.10	0.16	0.62
SOAP	11	15	62	118	0.08	0.09	1.11	1.63
MACE	8	17	21	29	0.06	0.06	0.11	0.27

In contrast, training on a smaller data set, i.e., the MP → Kraka case, where MP contains only 43 data points, fails to generalize well to a larger data set, which could also be related to limitations of the GPR method. To explore this, we employed different regressors as described in Section 2.4. It is known that, in terms of balance betwen accuracy and computational cost, BR regression is optimal to use with small data sets and our results confirm this for all descriptors tested here (Table 6); more detailed tables are provided in Section S4.

To further assess the efficiency of BR compared to GPR, we analyzed the improvement in predictions across descriptors. Table 6 shows the percentage improvement achieved with BR regression for frequency and NMR predictions using different descriptors. Figure S2 shows the frequency scatterplots for the GPR and BR regressions for the wACSF descriptor as an example. These results highlight the ability of BR regression to capture general trends in the data more effectively than GPR, particularly when training data is limited, offering notable advantages over GPR in specific contexts.

**Table 6:** Comparison of the Predictive Performance (RMSD Values) for ML Models Created Using Two Different Regressors: GPR and Bayesian Ridge Regressor (BR)^a^

	OH frequency with GPR or BR			Chemical shift with GPR or BR		
	MP → Kraka			MP → Kraka		
Descriptor	GPR regression RMSD (cm^–1^)	BR regression RMSD (cm^–1^)	GPR ⇒ BR ΔRMSD^b^,^c^	GPR regression RMSD (cm^–1^)	BR regression RMSD (cm^–1^)	GPR ⇒ BR ΔRMSD^b^,^c^
R(O···O)	67	62	2%	0.62	0.61	2%
R(H···O)	33	32	4%	0.60	0.61	–2%
Tetrahedron	37	36	3%	0.28	0.29	<±1%
R30(H···X)	80	44	60%	0.72	0.53	26%
R30(H···O)	102	40	76%	0.57	0.43	26%
PDF	146	57	61%	0.89	0.54	39%
LMBTR	73	46	34%	0.81	0.58	28%
ACSF	94	38	51%	0.57	0.41	28%
wACSF	171	31	65%	0.62	0.32	48%
SOAP	118	62	47%	1.63	0.43	192%
MACE	29	29	<±1%	0.27	0.27	<±1%

a The regressor comparison is made for all descriptors, with optimized hyperparameters, examined in this study, and we use MP → Kraka cross-validation (model from one data set is assessed with the other data set as the test set).

b Percentage improvement in going from GPR ⇒ BR is calculated as −[(RMSD_GPR_ – RMSD_BR_)/RMSD_GPR_]. Thus a positive value indicates improvement (lower RMSD) when going from GPR to BR.

c Although the RMSD values are displayed with 2 significant digits, the percentages were calculated using higher precision. |ΔRMSD| changes less than 1% are given the generic entry “<±1%”.

### 3.4 Data Set Size

An important characteristic of our fitting procedure is the significant difference in the number of data points between the two data sets. The Kraka data set contains approximately 23 times more data points than the MP data set. Therefore, it is interesting to consider the minimum number of data points required for successful model fitting. To investigate this, we used a data sampling method that ensures balanced selection of data points from the Kraka data set (details are given in Section S5). Using frequency as the target property, we varied the number of sampled data points used in a 5-fold splitting scheme.

Figure 4a,b shows the change in RMSD values for the MACE descriptor (Figure 4c,d is for the R­(H···O) descriptor) as a function of the number of training data points. The data points excluded from the training set were used as an additional external validation set (referred to as Kraka cross-validation). As shown, using approximately 300 data points from the Kraka data set is sufficient to achieve acceptable RMSD values, indicating that the MACE model performance saturates beyond this point. For the simpler R­(H···O) based models, less data is needed, as expected. This also explains why the MP → Kraka cross-validation is so challenging.

**Figure 4:**
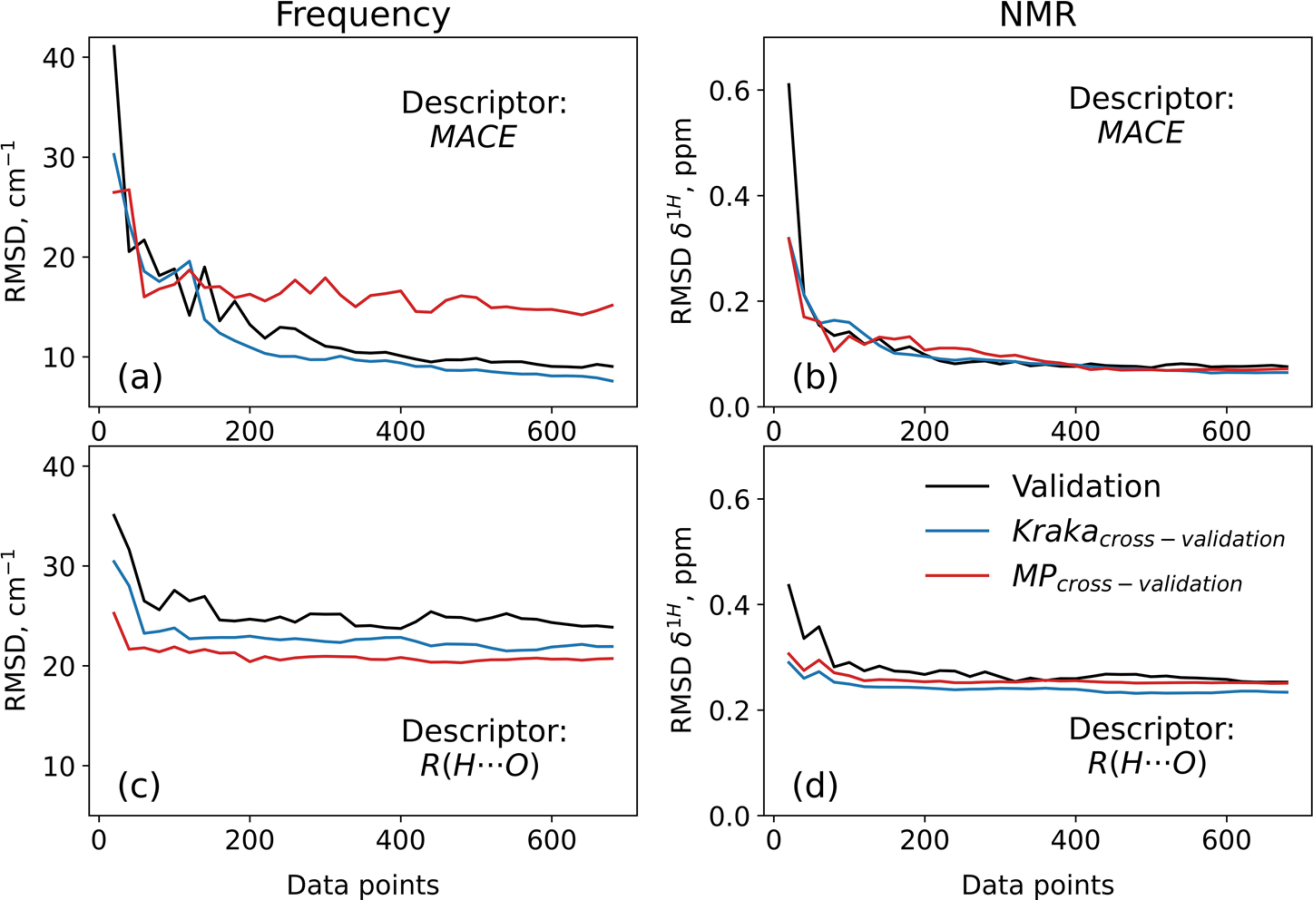
RMSD values for the Kraka-based fitted models, plotted as a function of the number of data points included in the training. The RMSD values originate from three different validation/testing schemes. The top panels (a,b) make use of the MACE descriptor, and the bottom panels (c, d) make use of the R­(H···O) descriptor. The color scheme of the graphs is explained in (d). “Validation” refers to our standard 5-fold validation procedure, but now involving only “folds” within the reduced training sets given on the x-axes. “Kraka cross-validation” means that the resulting models are validated on the remaining Kraka data, i.e. the Kraka data not used for training. "MP cross-validation" means that the resulting models are validated on the MP data. As the number of training data points increases, the RMSD values in the plots converge to those reported in Table 4.

### 3.5 Summary of Section 3

First, to achieve as consistent comparisons between descriptors as possible, we used the same structures, the same DFT method, consistent hyperparameters, and the same measures of quality in all comparisons, and both for the vibrational and the NMR models. This is how the models in Section 3.1 were constructed. Next, in Section 3.2, we optimized the hyperparameters to achieve the “best possible” prediction with each descriptor and regressor. In cases where data are limited, such as with the MP data set, the choice of regression model becomes important. The combination of an optimized descriptor with BR regression becomes a fast and effective solution due to its built-in regularization and probabilistic framework. We found that using a high number of radial and angular functions in the descriptor did not necessarily lead to better results; with a modest number of functions, it was possible to achieve very good performance. Thus, while a previous liquid water study employed up to 256 ACSF functions,^9^ we found that a reduced set of 16–32 ACSF functions was sufficient in our case. Furthermore, depending on whether we are dealing with the NMR shifts or the vibrations, the inclusion of (more) radial and/or angular parts in the descriptor could matter. For example, the chemical shifts are found to be more sensitive to the inclusion of angular terms than the vibrational frequencies.

## 4 Concluding Remarks

In this study, we numerically investigated key factors influencing model performance when predicting spectroscopic properties from atomic structures in ice structures. The universal MACE descriptor that we used performs remarkably well, with high accuracy for both the large and small data sets and for both spectral properties investigated here, effectively capturing complex local environments. At the same time, foundation models are computationally intensive to use and generally lack physical interpretability. To match the performance of MACE while reducing the computational use cost, we systematically optimized various atom-centered descriptors and regression models. As a result, we demonstrate that, for our systems, MACE-level accuracy can (almost) be achieved using simpler descriptors such as ACSF and SOAP when paired with suitable regressors, as was seen in Figure 3. For instance, in Kraka → MP cross-validation calculations with the SOAP descriptor we found RMSD values of ∼15 cm^–1^ for vibrational frequencies and ∼0.1 ppm for NMR shifts, which are comparable to those of the MACE model. These findings show the importance of balancing model complexity, data set scope, and computational cost when designing predictive tools in materials science.

Furthermore it should be noted that the ice data sets used in this work represent only a narrow region of the much larger and more diverse space of crystalline hydrates, some of which were considered in ref 22. For broad, multielement crystalline hydrate databases, advanced models like MACE or ShiftML for NMR shifts may be required to capture complex structural information of such systems. In contrast, for element-specific descriptors (e.g., ACSF and SOAP), the feature dimension grows combinatorically (approximately binomially) with the number of elements making them unfeasible in such situations. The simple R­(H···O) descriptor, on the other hand, is only applicable to systems where the hydrogen-bond acceptor is an oxygen atom. In our application, complex descriptors are not necessarily needed to achieve acceptable model quality as we use large, homogeneous, and high-fidelity data sets (the Kraka-UU NMR and frequency data sets). Choosing the right descriptor/regressor for the size and type of data set is important, especially if data or computational resources are limited. In our study, simple distance-based or atom-centered descriptors can be quite efficient and may be adequately predictive depending on the task at hand.

## Supplementary Material



## Notes

Data curation (Section S1)—description of data set preparation, cleaning, and structure validation procedures (Table S1); descriptor visualization (Section S2)—graphical representation of MACE features (Figure S1); tuning of hyperparameters (Section S3)—performance scores for different descriptor hyperparameters (Tables S2–S9); cross-validation (Section S4)—results for the MP → Kraka case using different regressors (Table S10) and scatter plots for wACSF GPR and BR models (Figure S2); data point selection (Section S5)—description of Poisson disk sampling for representative data selection (Figure S3) (PDF)
