# Supplementary material for: Machine-Learning Ice Spectra: From 1 to 256 Features
Source: J Chem Theory Comput. 2026 Feb 4;22(4):1834–45. doi: 10.1021/acs.jctc.5c01413 (PMC12937092; doi:10.1021/acs.jctc.5c01413)
Supplement: Supplementary file 1 [file ct5c01413_si_001.pdf]

# SUPPLEMENTARY INFO

# **Machine-learning ice spectra: from 1 to 256 features**

Shokirbek Shermukhamedov, Jolla Kullgren, Daniel Sethio, and Kersti Hermansson*

Department of Chemistry-Ångström, Uppsala University, Box 538, S-75231 Uppsala (Sweden)

*Corresponding-author:
E-Mail: *kersti@kemi.uu.se*

Contents

| | |
|---|---|
| Section S1: Data curation | p. S2 |
| Section S2: Descriptor visualization | p. S3 |
| Section S3: Tuning of hyperparameters | pp. S4-S6 |
| Section S4: Cross-validation | p. S7 |
| Section S5: Data point selection | p. S8 |

# Section S1: Data curation

Analysis of the MP dataset revealed the presence of structures with differently hydrogen-ordered ice polymorphs too, i.e. mp-558226, mp-557082 and mp-558958 represent the metastable proton-ordered form of Ice XIV. These three structures were excluded due to the anomalies in their structures. Additionally, three crystals representing bulk water were identified and excluded from the dataset. Furthermore, three ice crystal structures were omitted due to specific issues: Ice Ih (mp-673658) was excluded due to its dissociated form, while Ice II (mp-684678) and Ice V (mp-634812) were removed due to anomalies in their unit cells.

*More details about the various phases of our data curation procedure for the structures in the MP data set and the Kraka dataset are given* in *Section 2.1 of the main text.*

Table S1. Materials Project crystal IDs and corresponding ice forms used in the MP dataset.

| MP id | Form of Ice |
|---|---|
| mp-696735 | Ice ix<br>Ice iii |
| mp-697111 | water |
| mp-697085 | ice viii |
| mp-558226 | Ice xiv |
| mp-557082 | ice xiv |
| mp-32959 | Ice VII |
| mp-703459 | Low temperature ice |
| mp-558958 | ice xiv |
| mp-684704 | ice Ih |
| mp-24043 | Ice ii: |

# Section S2: Descriptor visualization

To visualize vector-array descriptors, we used Principal Component Analysis (PCA). PCA is a statistical technique that reduces the dimensionality of large feature spaces while preserving most of the variance in the data. This reduction helps reveal trends, similarities, and outliers in complex datasets, making it a valuable tool for interpreting model features. Figure S1 presents the two-component PCA projection of the MACE descriptor for the Kraka dataset.

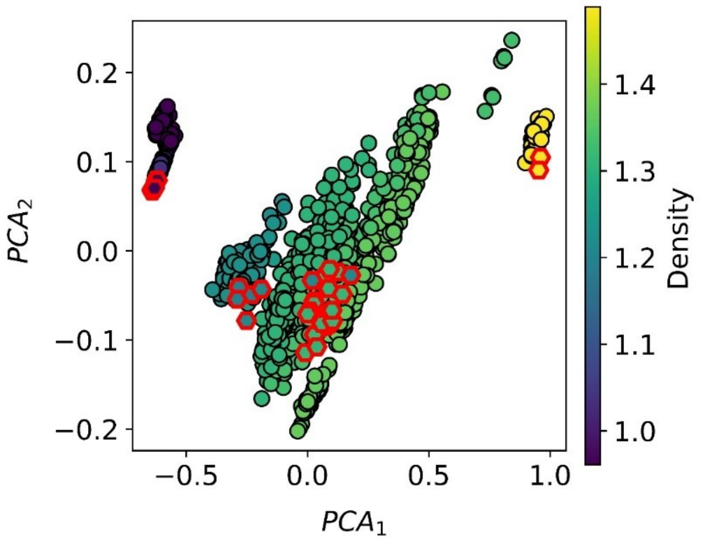


Figure S1.PCA analysis of the MACE feature space, supporting the findings presented in this study.

# Section S3: Tuning of hyperparameters

This section contains tables with the results of calculations for atomic-centered descriptor hyperparameter optimizations for the *Kraka→Kraka* case. All hyperparameter optimizations were performed in the same way as in the main calculations. For each optimization step, the descriptor hyperparameters were systematically varied to generate a new feature vector for every hydrogen atom, which was then used to fit the model using a Bayesian Ridge (BR) regressor. The goal of this procedure was to identify the optimal combination of descriptor parameters that minimizes the RMSD and improves model transferability within the Kraka dataset.

**Table S2**. RMSD values for OH vibrational frequency predictions (left) and $^1$H NMR chemical shift predictions (right) as a function of SOAP descriptor hyperparameters. $n_{max}$ denotes the number of radial basis functions, and $l_{max}$ represents the maximum angular degree of the spherical harmonics.

| | **Frequency** | | | | **NMR** | | | |
|---|---|---|---|---|---|---|---|---|
| | **$l_{max}$** | | | | **$l_{max}$** | | | |
| $n_{max}$ | 0 | 1 | 2 | 3 | 0 | 1 | 2 | 3 |
| 2 | 27 | 22 | 17 | 16 | 0.25 | 0.18 | 0.17 | 0.16 |
| 4 | 19 | 14 | 12 | 11 | 0.17 | 0.11 | 0.09 | 0.08 |
| 8 | 17 | 12 | 11 | 10 | 0.14 | 0.09 | 0.08 | 0.07 |
| 10 | 18 | 13 | 11 | 10 | 0.14 | 0.1 | 0.08 | 0.07 |
| 12 | 18 | 12 | 11 | 10 | 0.14 | 0.09 | 0.08 | 0.08 |
| 14 | 17 | 12 | 11 | 10 | 0.13 | 0.09 | 0.08 | 0.08 |
| 16 | 17 | 12 | 11 | 11 | 0.13 | 0.09 | 0.08 | 0.08 |

**Table S3**. RMSD values for OH vibrational frequency predictions as a function of the number of radial and angular atomic-centered symmetry functions.

| | Kraka → Kraka | | | | | | | |
|---|---|---|---|---|---|---|---|---|
| | Pairs of Angular ACSFs | | | | | | | |
| Radial ACSFs | 0 | 2 | 3 | 5 | 7 | 9 | 12 | 16 |
| 4 | 36 | 18 | 17 | 16 | 15 | 15 | 15 | 14 |
| 8 | 21 | 18 | 17 | 16 | 15 | 14 | 14 | 14 |
| 16 | 21 | 17 | 16 | 15 | 14 | 14 | 14 | 13 |
| 32 | 20 | 16 | 15 | 14 | 13 | 13 | 13 | 13 |
| 64 | 20 | 16 | 15 | 14 | 13 | 14 | 13 | 13 |

**Table S4**. RMSD values for $^{1}H$ NMR chemical shift predictions as a function of the number of radial and angular atomic-centered symmetry functions.

| | Kraka → Kraka | | | | | | | |
|---|---|---|---|---|---|---|---|---|
| | Pairs of Angular ACSFs | | | | | | | |
| Radial ACSFs | 0 | 2 | 3 | 5 | 8 | 10 | 12 | 16 |
| 4 | 0.36 | 0.17 | 0.16 | 0.12 | 0.11 | 0.11 | 0.11 | 0.11 |
| 8 | 0.21 | 0.15 | 0.14 | 0.11 | 0.11 | 0.11 | 0.1 | 0.11 |
| 16 | 0.25 | 0.14 | 0.13 | 0.12 | 0.11 | 0.11 | 0.11 | 0.11 |
| 32 | 0.30 | 0.13 | 0.13 | 0.12 | 0.11 | 0.1 | 0.1 | 0.11 |
| 64 | 0.30 | 0.13 | 0.13 | 0.12 | 0.11 | 0.11 | 0.1 | 0.11 |

**Table S5**. RMSD values for OH vibrational frequency predictions as a function of the number of radial and angular weighted atomic-centered symmetry functions.

| | Kraka → Kraka | | | | | | | |
|---|---|---|---|---|---|---|---|---|
| | Pairs of Angular ACSFs | | | | | | | |
| Radial ACSFs | 0 | 2 | 3 | 5 | 7 | 9 | 12 | 16 |
| 4 | 23 | 21 | 20 | 19 | 19 | 19 | 19 | 19 |
| 8 | 21 | 20 | 20 | 18 | 18 | 17 | 18 | 19 |
| 16 | 21 | 20 | 19 | 17 | 17 | 17 | 18 | 18 |
| 32 | 20 | 20 | 18 | 16 | 16 | 15 | 16 | 17 |
| 64 | 19 | 18 | 18 | 15 | 16 | 15 | 16 | 16 |

**Table S6**. RMSD values for $^{1}H$ NMR chemical shift predictions as a function of the number of radial and angular weighted atomic-centered symmetry functions.

| | | Kraka →Kraka | | | | | | |
|---|---|---|---|---|---|---|---|---|
| | | Pairs of Angular ACSFs | | | | | | |
| Radial ACSFs | 0 | 2 | 3 | 5 | 7 | 9 | 12 | 16 |
| 4 | 0.29 | 0.26 | 0.22 | 0.21 | 0.2 | 0.2 | 0.19 | 0.18 |
| 8 | 0.19 | 0.17 | 0.17 | 0.16 | 0.16 | 0.16 | 0.16 | 0.16 |
| 16 | 0.17 | 0.17 | 0.17 | 0.15 | 0.15 | 0.15 | 0.15 | 0.14 |
| 32 | 0.16 | 0.16 | 0.15 | 0.13 | 0.13 | 0.13 | 0.13 | 0.13 |
| 64 | 0.16 | 0.15 | 0.15 | 0.13 | 0.13 | 0.13 | 0.13 | 0.13 |

**Table S7**. RMSD values for OH vibrational frequency predictions (left) and $^1$H NMR chemical shift predictions (right) as a function of the number of radial grid points and scaling parameters used in the LMBTR descriptor.

| | **Frequency** | | | | **NMR** | | | |
|---|---|---|---|---|---|---|---|---|
| | **Scaling factor** | | | | **Scaling factor** | | | |
| $n_{max}$ | 0.01 | 0.1 | 0.2 | 0.5 | 0.01 | 0.1 | 0.2 | 0.5 |
| 10 | 28 | 22 | 18 | 20 | 0.32 | 0.22 | 0.15 | 0.21 |
| 30 | 27 | 16 | 16 | 18 | 0.28 | 0.12 | 0.11 | 0.15 |
| 50 | 23 | 16 | 16 | 18 | 0.24 | 0.11 | 0.11 | 0.15 |
| 75 | 20 | 16 | 16 | 18 | 0.21 | 0.11 | 0.11 | 0.15 |
| 100 | 18 | 16 | 16 | 18 | 0.18 | 0.11 | 0.11 | 0.15 |
| 200 | 19 | 16 | 16 | 18 | 0.2 | 0.11 | 0.11 | 0.15 |
| 400 | 20 | 16 | 16 | 18 | 0.2 | 0.11 | 0.11 | 0.15 |

**Table S8**. Dependence of the OH vibrational frequency RMSD on the number of radial grid points and scaling factors defining the PDF descriptor.

| | Kraka →Kraka | | | | | |
|---|---|---|---|---|---|---|
| | Scaling factor | | | | | |
| $n_{max}$ | 0.3 | 0.5 | 1 | 1.2 | 1.6 | 2 |
| 10 | 36 | 36 | 36 | 36 | 36 | 38 |
| 30 | 30 | 30 | 30 | 30 | 30 | 30 |
| 50 | 29 | 29 | 29 | 29 | 30 | 30 |
| 60 | 29 | 29 | 29 | 29 | 29 | 30 |
| 100 | 26 | 26 | 26 | 26 | 26 | 27 |
| 200 | 23 | 23 | 22 | 22 | 22 | 22 |

**Table S9**. Dependence of the NMR shifts RMSD on the number of radial grid points and scaling factors defining the PDF descriptor

| | Kraka →Kraka | | | | | |
|---|---|---|---|---|---|---|
| | Scaling factor | | | | | |
| $n_{max}$ | 0.3 | 0.5 | 1 | 1.2 | 1.6 | 2 |
| 10 | 0.41 | 0.41 | 0.41 | 0.41 | 0.41 | 0.42 |
| 30 | 0.29 | 0.29 | 0.29 | 0.29 | 0.3 | 0.3 |
| 50 | 0.27 | 0.27 | 0.27 | 0.27 | 0.28 | 0.29 |
| 60 | 0.26 | 0.26 | 0.26 | 0.26 | 0.27 | 0.27 |
| 100 | 0.24 | 0.24 | 0.23 | 0.23 | 0.24 | 0.24 |
| 200 | 0.2 | 0.2 | 0.19 | 0.19 | 0.19 | 0.19 |

# Section S4: Cross-validation

**Table S10.** Comparison of the predictive performance for ML models that were created using a range of different regressors on the MP dataset and with the MACE descriptor.

| Regressor | | OH frequency with MACE MP→Kraka | | | [1]H chemical shift with MACE MP→Kraka | | |
|---|---|---|---|---|---|---|---|
| | | RMSD | $R^2$ | AMD | RMSD | $R^2$ | AMD |
| **Linear** | Linear | 31 | 0.83 | 51 | 0.99 | -2.49 | 3.28 |
| | Ridge | 36 | 0.77 | 73 | 0.57 | -0.02 | 0.82 |
| | BR | 29 | 0.85 | 73 | 0.27 | 0.76 | 0.63 |
| **Prob.** | GPR | 29 | 0.86 | 65 | 0.27 | 0.77 | 0.49 |
| **NN** | MLP | 454 | -45.9 [a] | 1048 | 1.19 | -3.85 | 3.02 |
| | CONV | 102 | -0.79 | 127 | 0.86 | -1.36 | 0.75 |
| | LSTM | 90 | -0.48 | 237 | 0.61 | -0.19 | 0.74 |

[a)] The MLP completely failed to fit the frequencies.

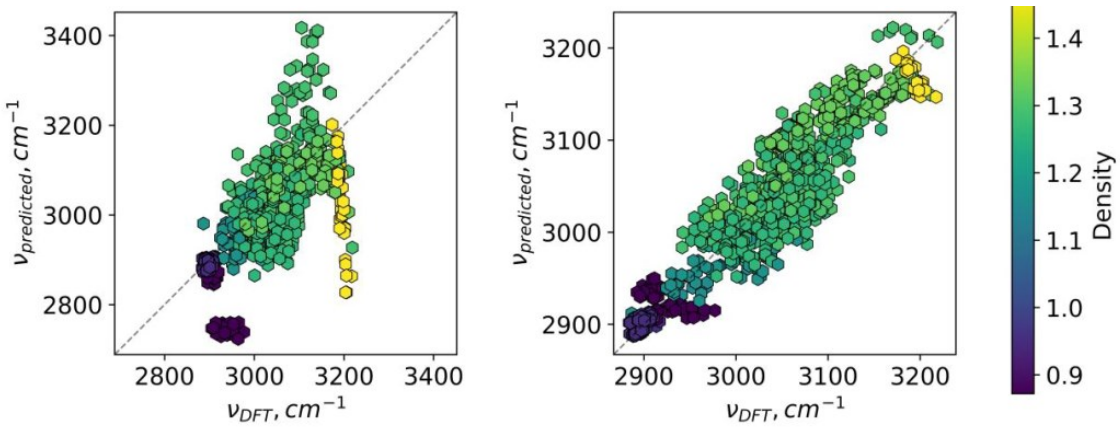


Figure S2. Scatter plots of reference versus predicted frequencies using Kraka data as the test set, showing predictions from the MP-trained model with the wACSF descriptor and GPR (left) and BR (right) regressors.

# Section S5: Data point selection.

We used Poisson disk sampling rather than random selection to ensure the identification of relevant data points. This method chooses data points in a way that prevents clustering and keeps them evenly distributed. Unlike random sampling, it enforces a minimum distance between any two chosen locations, ensuring better coverage of the feature space. This makes it especially useful for high-dimensional datasets, where consistent spacing improves representation and reduces duplication. Poisson disk sampling is widely used for effective and unbiased data point selection in scientific simulations, computer graphics, and machine learning. Figure S3 shows an example of selecting different numbers of data points.

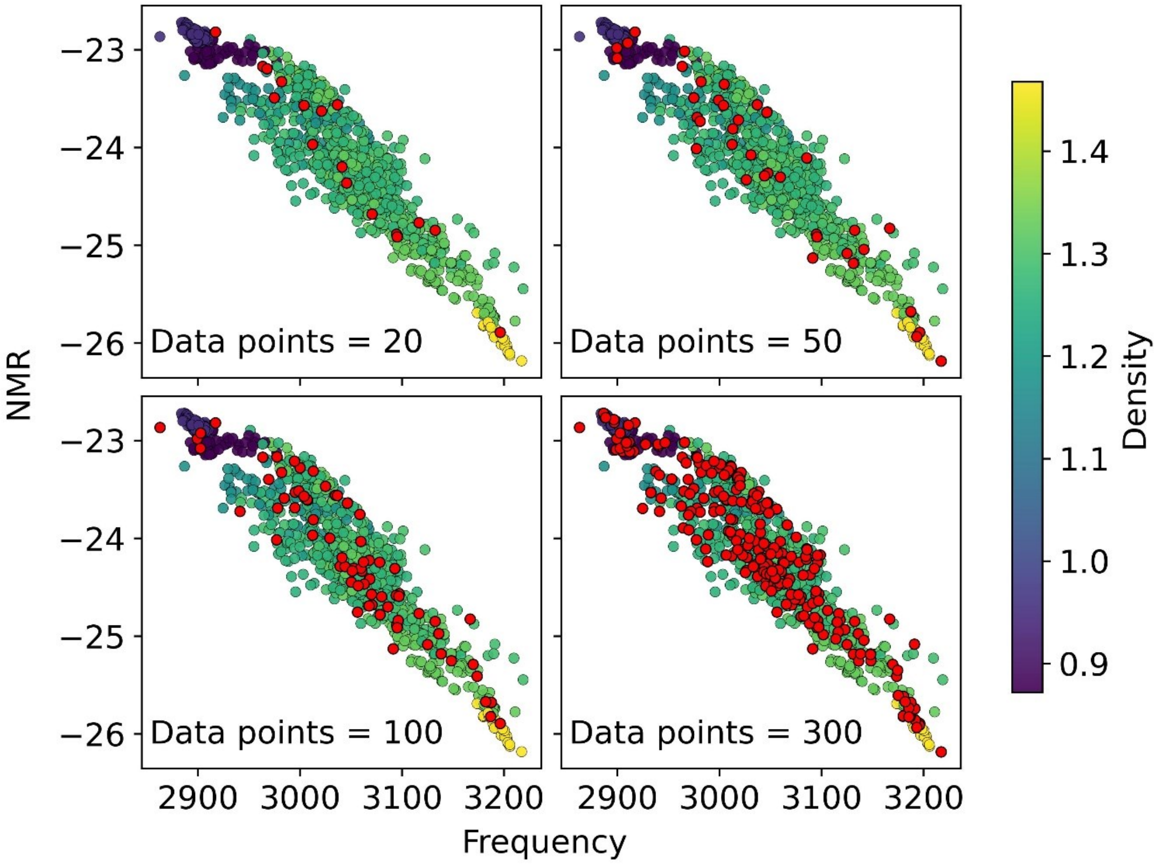


Figure S3. Example of data point selection (red circles) using Poisson disk sampling. Different numbers of data points are shown to illustrate how the method maintains uniform spacing and avoids clustering across the feature space.
